# The Effect of Endometrial Polyp and Myoma Uteri on Fertility-Related Genes in the Endometrium

**DOI:** 10.1007/s43032-025-01802-0

**Published:** 2025-02-06

**Authors:** Gürkan Özbey, Görkem Tuncay, Senem Arda Düz, Yılmaz Çiğremiş, Abdullah Karaer

**Affiliations:** 1 Department of Obstetrics and Gynecology, Faculty of Medicine, Adıyaman University, Adıyaman, Türkiye; 2 Department of Obstetrics and Gynecology, Faculty of Medicine, Inonu University, Malatya, Türkiye; 3 Department of Medical Biology and Genetics, Faculty of Medicine, Inonu University, Malatya, Türkiye

**Keywords:** Myoma uteri, Endometrial polyp, Infertility, Gene expression, *HOXA10*

## Abstract

Endometrial polyps are hyperplastic overgrowths of the endometrium that contain both glands and stroma. Myoma uteri is the most common benign tumor of the female pelvis and uterus. *HOXA10*, which is involved during the organogenesis of the uterus in the embryonic period. The aim of this study was to compare the expression levels of infertility-related genes in endometrial tissue obtained from patients with endometrial polyp and myoma uteri and from healthy controls. A total of 36 patients, including 15 women with endometrial polyp and 21 women with myoma uteri, and 23 healthy controls were enrolled in the study. All patients were evaluated using transvaginal ultrasonography. Endometrial tissue samples were collected from the patient and control groups between the 19th and 21st days of the menstrual cycle. Expression levels of the receptivity markers *PROK1, PROKR1, PROK2, PROKR2* and *HOXA10* genes were determined by RT- PCR. When the patients diagnosed with endometrial polyp and the healthy controls were compared, it was observed statistically significantly that the expression of *PROKR1* increased in endometrium tissue of patients with endometrial polyp (*p* < 0.05). In patients diagnosed with myoma uteri, gene expression levels of endometrial PROKR1 was statistically significant increased and gene expression levels of *PROK1, PROKR2, HOXA10* were found to be statistically significantly decreased compared to the controls (*p* < 0.05). Changes in the endometrial expression of the *HOXA10* and prokineticin gene family in patients with myoma uteri and endometrial polyps may explain certain aspects of infertility in these patients.

## Introduction

Endometrial polyps (EP) are predominantly benign overgrowths of the endometrial lining, commonly occurring in women of reproductive age and peaking in prevalence during the fifth decade of life [1]. These polyps are typically identified through endometrial biopsies or hysterectomies, with detection rates varying between 10 and 24% in clinical samples and 8–10% in autopsy specimens [2]. Among asymptomatic women seeking infertility treatment, the prevalence rises to 15%, suggesting a potential association between EP and infertility [3]. Interestingly, polyps measuring 0.7 to 1.3 cm in premenopausal women may regress spontaneously [4], highlighting the complex and dynamic nature of their clinical course. While most EPs are benign, some exhibit premalignant or malignant transformations, particularly in the context of endometrial hyperplasia, underscoring the need for a deeper understanding of their molecular basis and potential implications for fertility [1]. Diagnostically, EPs are most accurately identified through hysteroscopy, the gold standard, although non-invasive techniques such as transvaginal ultrasonography, sonohysterography, and saline infusion sonography are also widely used [5]. Morphologically, EPs can present as sessile or pedunculated, singular or multiple, and may co-occur with cervical polyps. Clinically, they manifest with a spectrum of symptoms, ranging from menstrual irregularities to asymptomatic presentations, often detected incidentally during infertility evaluations. Notably, EPs are identified in approximately 25% of patients undergoing hysteroscopy for unexplained infertility [6]. It is essential to distinguish EPs from other uterine pathologies such as myoma uteri, the most common benign uterine tumor, which affects 20–77% of premenopausal women and accounts for 30% of hysterectomies [1]. Recent studies suggest a potential overlap in the genetic and molecular pathways underlying EP and myomas, warranting further investigation into their shared mechanisms [7]. Among the genes implicated in endometrial receptivity and implantation, *HOXA10* and *PROKR1* stand out as critical regulators of uterine function. HOXA10, a transcription factor within the HOX gene family, plays a pivotal role in reproductive tract development and adult endometrial receptivity. It promotes implantation by regulating the expression of key adhesion molecules, such as integrins, and growth factors essential for embryo attachment and survival [8–10].

*HOXA10* expression peaks during the mid-luteal phase, coinciding with the implantation window, and is hormonally regulated, further underscoring its role in establishing a receptive endometrial environment [8, 11, 12]. Dysregulation of HOXA10 has been associated with infertility, recurrent pregnancy loss, and poor outcomes in assisted reproductive technologies (ART) [13, 14]. *PROKR1*, the receptor for prokineticin-1 (*PROK1*), is integral to angiogenesis, inflammatory regulation, and vascular remodeling in the endometrium. It mediates processes essential for preparing the endometrium for implantation, including vascularization and immune cell recruitment [15]. The expression of *PROKR1* and its ligand, *PROK1*, is upregulated during the luteal phase and early pregnancy, highlighting their critical role in uterine receptivity [16–18]. Furthermore, PROK1 signaling, modulated by endocrine gland-derived vascular endothelial growth factor (EG-VEGF), is crucial for maintaining tissue-specific angiogenesis and endometrial function [15]. Emerging evidence suggests that *PROK1* serves as a marker for uterine receptivity, supporting successful implantation and early pregnancy maintenance [19–21]. The interplay between HOXA10 and PROKR1 highlights their combined importance in reproductive health. While HOXA10 facilitates structural and cellular preparations, PROKR1 enhances vascular and immune functionality, creating a synergistic framework for optimal implantation. Moreover, PROK1 and its receptors (PROKR1 and PROKR2) are G protein-coupled receptors (GPCRs) that mediate intracellular signaling pathways essential for reproductive function [15, 22–24]. These receptors, encoded on chromosomes 2p13.1 and 20p12.3, share high amino acid homology and play a crucial role in regulating vascularization and immune responses in the endometrium. Their activity is particularly relevant in the context of fertility, as they regulate angiogenesis and inflammatory pathways that are essential for the establishment of a successful pregnancy. This intricate coordination underscores their relevance in fertility and provides a basis for exploring targeted interventions in infertility treatments. This research could offer valuable insights into the molecular basis of uterine pathologies and help inform future fertility treatments.

This study investigates the expression levels of HOXA10, PROKR1, PROK1, and related genes in endometrial tissues from patients diagnosed with EP and myoma uteri. By comparing these expression patterns with those in healthy controls, this research aims to elucidate the molecular mechanisms underlying these uterine pathologies and their impact on fertility. These findings could contribute to advancing diagnostic and therapeutic approaches for infertility and related disorders.

## Material and Methods

### Subjects

This study was planned as a prospective case–control study. A total of 36 patients, including 15 patients with histopathological diagnosis of endometrial polyps and 21 patients with myoma uteri, who applied to the Inonu University Faculty of Medicine, Department of Obstetrics and Gynecology between June 2019 and November 2020, were included in this study. Twenty-three patients without endometrial polyp and myoma uteri pathology were included in the control group. A standard form was prepared and the information and physical examination findings of each patient and control group were recorded. As the patient group inclusion criteria; The patient is between 20–49 years old, between the 19th and 21st days of the menstrual cycle, it is determined by hysteroscopy or saline infusion sonography or transvaginal USG that there is an endometrial polyp or myoma uteri in the uterine cavity, and the diagnosis of endometrial polyp or myoma uteri is confirmed histopathologically after surgery. The control group inclusion criteria are; The controls were between 20 and 49 years of age, between the 19th and 21st days of the menstrual cycle, no history of infertility, no evidence of endometrial polyp and myoma uteri by imaging methods, no finding of endometrial polyp or myoma uteri in the endometrial sampling results. Age, gravida, parity, abortion and number of living, chronic disease, education level, birth control method used, if any, and infertility duration of the patients included in the study were questioned. Patients who were outside the age range of 20 to 49 years, were not in the reproductive period, had a history of previous myomectomy or polypectomy, and were outside the 19–21 days of the menstrual cycle were not included in the study. After the bladder was emptied in the lithotomy position after the gynecological examination, ultrasonographic examinations of all patients were performed by a single clinician using the IC5-9-D 7 MHz transducer of the Voluson (GE Healthcare, Milwaukee, WI, USA) ultrasonography device. Patients with suspected endometrial polyps and myoma uteri were tried to confirm the diagnosis by performing saline infusion sonography or hysteroscopy or transvaginal USG. The diagnosis of endometrial polyp was made by hysteroscopy performed after transvaginal ultrasonography. The obtained materials were sent for pathological evaluation, and the diagnosis of polyps was confirmed histologically. All study participants were included in the study after obtaining a signed informed consent form after explaining the purpose and content of the study. The study was designed and performed in accordance with the Declaration of Helsinki, and was approved by the local ethics review board (approval number: 2019/77).

### The Samples

Endometrial tissue samples from patients with myoma uteri diagnosis and control group were taken by probe curettage (P/C), and endometrial samples from patients diagnosed with endometrial polyp were taken between the 19th and 21st days of the menstrual cycle by hysteroscopy. Then the samples were divided into two groups. First, samples were stored in 1 ml RNA-later solution at −80 ℃ until assay for real-time quantitative polymerase chain reaction (RT- PCR) and second the they were fixed in 10% buffered formalin for immunohistochemistry analysis. The timing of the biopsy was dated according to the last menstrual period indicated and was confirmed by histological evaluation according to the criteria set by Noyes et al. and Karaer et al. [24, 25].

### Clinical Measurement

#### RNA Extraction / cDNA Synthesis Protocol

Total RNA was extracted using the RNeasyJ Plus Mini Kit (Qiagen INC, Germantown, MD, USA) according to the manufacturer’s guidelines. For the reverse transcription process (RT^2^), the cDNA transcription kit RT^2^ kit, produced by Qiagen, was used. cDNA synthesis was performed in accordance with the protocol proposed by the company. In summary, 2.0 µg total RNA, 1 µl primer (4 pmol genspecific primer), 1 µl dNTP (10 mM) and bidistilled water were added to a 100 µl PCR tube with a total volume of 14 µl, mixed and heated in a PCR device at 65 °C for 15 min. 4 µl 5 × first strand buffer, 2 µl DTT, 1 µl distilled water, 1 µl RT2 reverse transcriptase enzyme were added to this mixture and mixed and heated in the PCR device at 50 °C for 60 min and then at 70 °C for 15 min, then stored at −20 °C until analyzed [24].

#### Real-Time PCR Protocol (RT-PCR)

As a real-time PCR device (RT-PCR), the analysis was performed on the Qiagen Rotorgene Q (Qiagen, Hilden) model. SYBR Green Master Mix (Qiagen) was used. The reactions were performed in a total volume of 25 µl and the Rotor-Disc was carried out in 72 discs. The mixture consisted of 5 µl cDNA, 1 µl advanced primer (10 pmol/ul), 1 µl reverse primer (10 pmol/ul), 6.5 uL DNase/RNase-free distilled water and 12.5 µl 2 × SYBR Green Master Mix. PCR conditions after optimization of primers; initial denaturation; 2 min at 95 °C, denaturation at 94 °C for 15 s, annealing at 60 °C for 30 s, and the number of cycles was 40 [24]. ACTB was used as the reference gene. The GenBank sequences for the respective primers are given in Table 1. In summary, Table 1 presents key genes involved in fertility and reproductive processes, with a focus on prokineticins, their receptors, and the important HOXA10 gene, all of which are integral to understanding infertility and implantation mechanisms. These genes are likely analyzed in relation to their expression levels in conditions such as endometrial polyps and myoma uteri.

**Table 1:** Human gene (RT^2^ primer assays qiagen)

Position	Ref Seq Number	Symbol	Description
1	NM_032414	PROK1	Prokineticine 1
2	NM_138964	PROKR1	Prokineticine receptor 1
3	NM_021935	PROK2	Prokineticine 2
4	NM_144773	PROKR2	Prokineticine reseptor 2
5	NM_018951	HOXA-10	Homeobox A10
6	NM_001101	ACTB	Actin beta

### Statistical Analysis

The data were analyzed using version 26.0 of the Statistical Package for the Social Sciences (SPSS) software for Windows (IBM/SPSS, Inc.). To assess the distribution of the data, the Kolmogorov–Smirnov test was applied. The age variable was presented as the mean ± standard deviation (SD) for both the patient and control groups. A p-value of less than 0.05 (*p* < 0.05) was considered statistically significant. The sample size was calculated using the G*Power analysis tool (version 3.1, Heinrich-Heine-University Düsseldorf, Düsseldorf, Germany). Based on a significance level (alpha) of 0.05 and a statistical power (1-beta) of 0.80, the analysis evaluated the impact of gene expression in endometrial tissue on fertility in patients with endometrial polyps and myoma uteri. The analysis anticipated an average difference of 0.97 units in HOXA10A mRNA expression between the patient groups and the control group. As a result, the required minimum number of participants per group was determined to be 21. Consequently, the study included at least 36 participants in the case groups and 23 in the control group (15 for the endometrial polyp group, 21 for the myoma uteri group, and 23 healthy controls), ensuring 80% power with an alpha of 0.05.

## Results

### Demographic Findings

A total of 36 patients, comprising 15 (25.4%) diagnosed with endometrial polyps (EP) and 21 (35.5%) diagnosed with myoma uteri, along with 23 (38.9%) healthy controls, participated in this study. The mean age of the EP patients was 38.07 ± 6.57 years, while the mean age of the myoma uteri patients was 42.67 ± 3.83 years. The mean age of the control group was 37.65 ± 6.03 years, and the mean age of the study group was 40.75 ± 5.56 years. A statistically significant difference was found between the mean age of the myoma uteri patient group and the control group (*p* = 0.01).

### The Gene Expression Levels of PROK1, PROKR1, PROK2, PROKR2, HOXA10 and ACTB in the Endometrial Tissue of Patients with Endometrial Polyps and Control Groups

*PROK1, PROKR1, PROK2, PROKR2*, *HOXA10* and *ACTB* gene expression levels between patients diagnosed with endometrial polyps and control groups are presented in Table 2. PROK1 (Fold change = 1.04, *p* = 0.827463): There is no significant difference in the expression of PROK1 between women with endometrial polyps and the control group (*p* > 0.05). The fold change is very close to 1, indicating a minimal difference in expression levels. *PROKR1 (Fold change = 8.66, *p* = 0.001925)**: A significant increase in PROKR1 expression is observed in women with endometrial polyps compared to the control group (*p* < 0.05). This suggests that PROKR1 may play a role in the pathology of endometrial polyps, potentially affecting fertility or implantation processes. PROK2 (Fold change = 5.91, *p* = 0.136303): There is an increase in PROK2 expression, but the difference is not statistically significant (*p* > 0.05). While there is a notable change, the result may not be strong enough to suggest a clear relationship with endometrial polyps. *PROKR2 (Fold change = 0.37, *p* = 0.0054276)**: PROKR2 shows a significant decrease in expression in women with endometrial polyps compared to controls (*p* < 0.05). This suggests a potential dysregulation of the prokineticin signaling pathway in the presence of polyps. HOXA10 (Fold change = 0.55, *p* = 0.149883): There is a decrease in HOXA10 expression in women with endometrial polyps, but this change is not statistically significant (*p* > 0.05). The result suggests that while there is a change in expression, it may not be robust enough to influence implantation or fertility. ACTB (Fold change = 1.00, *p* = non): As a housekeeping gene, ACTB serves as a control, and its fold change remains at 1.00, indicating consistent expression across all samples. The p-value is not applicable here.

**Table 2:** Comparison of *PROK1, PROKR1, PROK2, PROKR2* and *HOXA10* mRNA expression between women with Endometrial polyps and controls (fold change)

	Group 1 (Endometrial polyps)	Fold change (comparing to control group)	
Position	Gene Symbol	Fold change	P* value
1	PROK1	1.04	0.827463
2	PROKR1	8.66	0.001925*
3	PROK2	5.91	0.136303
4	PROKR2	037	0.0054276
5	HOXA10	0.55	0.149883
6	ACTB	1.00	non

*p < 0.05

The results indicate that PROKR1 and PROKR2 exhibit significant changes in expression between women with endometrial polyps and controls, with PROKR1 being upregulated and PROKR2 downregulated. These alterations suggest potential disruption of prokineticin signaling in endometrial polyps, which may impact fertility-related processes. However, no statistically significant changes were observed in PROK1, PROK2, and HOXA10, highlighting the need for further research to investigate their roles.

**Fig. 1:**
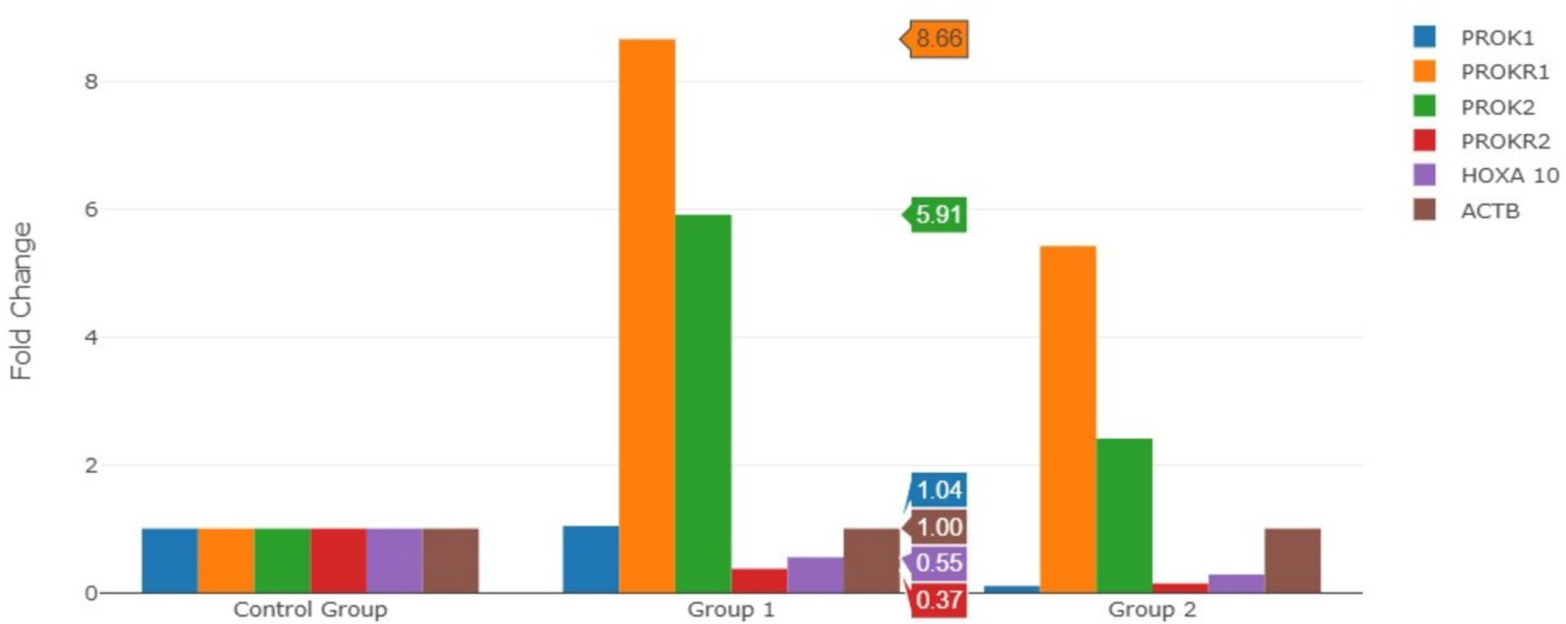
The Comparison of fold changes in the expression levels of PROK1, PROKR1, PROK2, PROKR2, HOXA10 and ACTB genes in control groups and patients diagnosed with endometrial polyps

No significant differences were found between the two groups in the expression levels of PROK1, HOXA10, and ACTB genes (Fig. 1).

#### Group 1

Group of patients diagnosed with endometrial polyps.

#### Control Group

Overall, the figure suggests that while PROKR1 and PROKR2 show the most significant changes, other genes like PROK1, PROK2, and HOXA10 exhibit smaller or non-significant alterations, highlighting the importance of certain prokineticins in the pathology of endometrial polyps.

### Gene Expression Levels of PROK1, PROKR1, PROK2, PROKR2, HOXA10 and ACTB in the Endometrial Tissue of Patients with Myoma Uteri and Control Groups

The gene expression levels of PROK1, PROKR1, PROK2, PROKR2, HOXA10, and ACTB in the endometrial tissue of patients diagnosed with myoma uteri and the control group are presented in Table 3.

**Table 3:** Comparison of *PROK1, PROKR1, PROK2, PROKR2* and *HOXA10* mRNA expression between women with myoma uteri and controls (fold change)

	Group 2 (Myoma uteri)	Fold change (comparing to control group)	
Position	Gene Symbol	Fold change	P* value
1	PROK1	0.10	0.026141
2	PROKR1	5.42	0.010739
3	PROK2	2.41	0.565634
4	PROKR2	0.14	0.001909
5	HOXA10	0.28	0.004209
6	ACTB	1.00	non

*p < 0.05

**Fig. 2:**
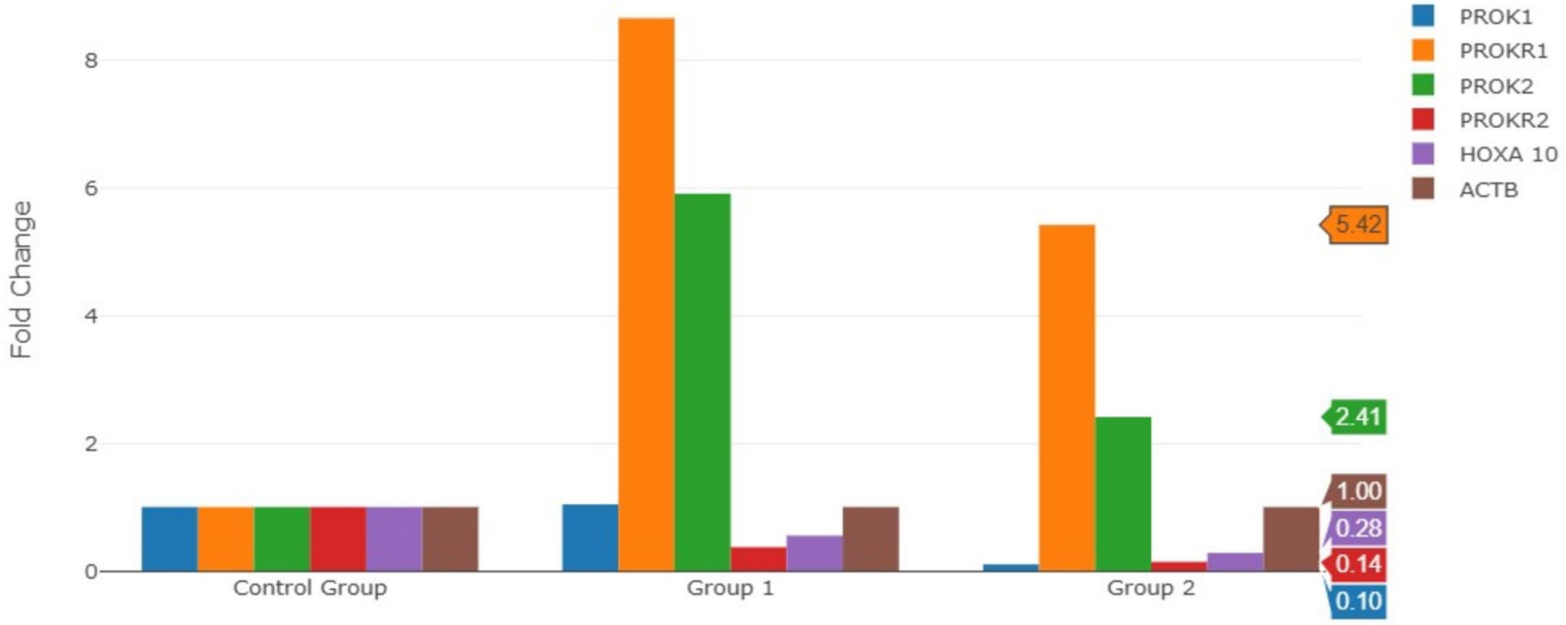
The Comparison of fold changes in the expression levels of PROK1, PROKR1, PROK2, PROKR2, HOXA10 and ACTB genes in control groups and patients diagnosed with myoma uteri

In this study, it was observed that the expression level of PROKR1 in patients diagnosed with myoma uteri increased 5.42 times compared to controls (*p* = 0.010739). The expression levels of PROK1, PROKR2, and HOXA10 were significantly decreased, with PROK1 at 0.10-fold, PROKR2 at 0.14-fold, and HOXA10 at 0.28-fold (*p* = 0.026141, *p* = 0.001909, and *p* = 0.004209, respectively). These differences were statistically significant (*p* < 0.05). The mRNA levels of PROK2 were 2.41 times higher (*p* = 0.565634) in women with myoma uteri compared to controls, but this increase was not statistically significant (*p* > 0.05) (Fig. 2).

#### Group 2

Group of patients diagnosed with myoma uteri.

#### Control Group

Figure 2 shows the comparison of gene expression changes between the control group and patients with myoma uteri (Group 2). Overall, the figure shows that significant changes in PROK1, PROKR1, PROKR2, and HOXA10 expression levels are observed in myoma uteri patients. Specifically, PROK1 and PROKR2 are downregulated, while PROKR1 is upregulated. These findings suggest that prokineticin signaling may be disrupted in myoma uteri, potentially influencing fertility-related processes. Further research is needed to confirm these results and explore the underlying mechanisms.

These findings suggest that PROK1 expression may be downregulated in the presence of myoma uteri. Additionally, the upregulation of PROKR1 indicates its potential involvement in the pathophysiology of myoma uteri. The downregulation of PROKR2 suggests a possible disruption of the prokineticin signaling pathway, while the observed decrease in HOXA10 expression may be linked to myoma uteri and could influence fertility. Collectively, these findings imply that changes in the expression of PROK1, PROKR1, PROKR2, and HOXA10 could play a role in the pathogenesis of myoma uteri, potentially impacting fertility. Further research is required to investigate the underlying mechanisms and clinical implications.

## Discussion

In this study, 36 patients were included, comprising 15 diagnosed with endometrial polyps (EP), 21 with myoma uteri (MU), and 23 healthy controls. All patients were evaluated using transvaginal ultrasonography (USG), and endometrial tissue samples were collected from both the patient and control groups. Patients with EP underwent hysteroscopic polypectomy, and tissue samples were obtained between the 19th and 21st days of the menstrual cycle. Gene expression levels of key receptivity markers—PROK1, PROKR1, PROK2, PROKR2, HOXA10, and ACTB—were determined using RT-PCR and analyzed using the Kolmogorov–Smirnov test. The study aimed to assess potential differences in the expression levels of infertility-related genes (HOXA10 and the prokineticin gene family) between the endometrial tissue of healthy controls and patients diagnosed with endometrial polyps or myoma uteri. A statistically significant increase in PROKR1 gene expression was observed in the endometrial tissue of both the EP and MU groups. However, no significant differences were found between the two patient groups in terms of the expression levels of other genes. Specifically, PROK1, PROKR2, and HOXA10 gene expressions were significantly decreased in the endometrial tissue of patients diagnosed with myoma uteri. No significant differences were found in the expression levels of PROK2 and ACTB. These results suggest that both endometrial polyps and myomas can induce changes in the gene expression of HOXA10 and prokineticins, which are associated with infertility, in the endometrium.

Endometrial polyps are benign tumors that arise from the endometrial lining and are typically attached by a stalk. Histologically, they resemble endometrial tissue, with central blood vessels and surrounding glandular hyperplasia. While the effects of asymptomatic endometrial polyps on fertility are not fully understood, these polyps have been shown to contribute to infertility by disrupting sperm and embryo transport, hindering embryo attachment, or reducing endometrial receptivity. The number, size, and location of polyps may also influence reproductive outcomes [26]. Despite some conflicting findings, several studies suggest that polyps are more frequently found in young infertile women and may contribute to infertility [21, 22, 27, 28]. The lack of significant change in HOXA10 expression in the endometrial tissue of women with EP in this study supports the idea that mechanical effects and local factors, rather than changes in genetic expression, may primarily mediate the relationship between polyps and infertility. Myoma uteri are the most common benign masses in women of reproductive age, with a higher prevalence in infertile women [29]. Approximately 5–10% of patients presenting with infertility have one or more fibroids [29, 30]. Studies have shown that the cumulative pregnancy rate in women with fibroids decreases by 40–50% over a one-year period [31, 32]. Besides the presence of myoma uteri, factors such as the size and number of fibroids play a critical role in fertility [33]. The location of fibroids is particularly important, with submucosal fibroids having the most significant impact on fertility [34]. Previous studies have demonstrated that myoma uteri most prominently affect fertility by impairing implantation [34, 35]. The HOXA10 gene plays a key role in uterine development and endometrial receptivity. Its expression, regulated by estrogen and progesterone, peaks during the midluteal phase, corresponding to the implantation window. This suggests that HOXA10 is involved in implantation and could serve as a potential marker for uterine receptivity [36]. HOXA10 regulates transcriptional expression by binding to regulatory regions of downstream target genes involved in endometrial development [37]. Impaired endometrial receptivity is a major cause of implantation failure, contributing to about two-thirds of implantation failures in infertile women [38, 39]. The impaired expression of HOXA genes has been observed in unexplained infertility, polycystic ovary syndrome (PCOS), endometriosis, and recurrent pregnancy loss [13, 40, 41], highlighting their role in embryo implantation. In the current study, no statistically significant difference in HOXA10 expression was found when comparing patients with endometrial polyps to healthy controls. This suggests that polyps may have a lesser effect on HOXA10 expression compared to myoma uteri. However, a significant decrease in HOXA10 expression was found in the endometrial tissue of patients diagnosed with myoma uteri.

The role of PROK1 in endometrial receptivity has been further emphasized by recent studies linking its expression to successful embryo implantation, particularly in IVF patients [19, 42]. PROK2 is mainly expressed in the central nervous system and nonsteroidogenic cells of the testes [43, 44]. Both PROK1 and PROK2 are expressed in several organs, including the brain, ovaries, testes, placenta, adrenal cortex, peripheral blood cells, intestines, heart, and bone marrow [45, 46]. PROK1 is primarily expressed in steroidogenic organs such as the ovaries, testes, adrenal cortex, and placenta [16], and it plays a role in regulating gonadal function in human reproduction [23]. In contrast, PROK2 has not been detected in human ovarian tissue [43]. PROK1 is also expressed in endometrial tissue, with its maximum expression levels occurring during the "implantation window" [46, 47]. Unlike HOXA10, this study observed a marked decrease in PROKR1 expression in the endometrium of women with recurrent implantation failure (RIF) at both the mRNA and protein levels. This finding aligns with previous research suggesting that PROKR1 plays a critical role in endometrial receptivity and implantation success [48–51]. Although PROK1 mRNA levels were significantly elevated in women with RIF compared to controls, this difference was not verified at the protein level. No significant differences were observed in PROK2 and PROKR2 expression between women with RIF and controls. The PROK1/PROKR1 signaling system coordinates the expression of key genes involved in implantation. Polymorphisms in the PROK1 and PROKR1 genes have been associated with recurrent miscarriage [15]. The expression of PROKR1 increases on the 4th day before implantation, decreases on the 5th day of implantation, and increases again from the 7th day post-implantation, indicating its involvement in processes essential for a healthy pregnancy. The expression of PROK1 and PROKR1 proteins before, during, and after implantation highlights their significant roles during these periods. In conclusion, this study identified a statistically significant increase in PROKR1 expression in the endometrial tissue of patients diagnosed with endometrial polyps and myoma uteri. Additionally, significant decreases in PROK1, PROKR2, and HOXA10 gene expression were observed in the endometrial tissue of patients with myoma uteri. These findings align with previous studies suggesting a correlation between polymorphisms in PROK1 and PROKR1 genes and recurrent miscarriage [15]. The impaired expression of HOXA10 and prokineticins is associated with conditions like unexplained infertility, PCOS, endometriosis, and recurrent pregnancy loss. Although HOXA10 and the prokineticin gene family are known to be involved in infertility, the exact mechanisms by which these genes influence fertility are not fully understood. Further research is needed to clarify their roles in the normal implantation process. Expanding knowledge of implantation processes in conditions like myoma uteri and endometrial polyps, as well as their connection to infertility, could offer valuable insights. Such findings may guide the development of new treatment strategies to improve implantation success in both spontaneous and assisted reproductive treatments. In the future, gene therapy may provide a more targeted treatment option, potentially increasing pregnancy rates by reducing implantation failure, a key cause of infertility in both spontaneous and IVF treatments, especially when related to endometrial factors.

## Conclusion

This study was conducted at the Department of Obstetrics and Gynecology, Inonu University Faculty of Medicine, between June 2019 and November 2020, with a total of 36 patients. Although the study involved 36 patients and 23 controls, the use of molecular analyses (RT-PCR) and comparisons made with carefully selected patients significantly mitigate the impact of this limitation. RT-PCR is a robust method that accurately measures gene expression and allows for the precise analysis of changes in endometrial tissue. Additionally, the minimum sample size was determined through G*Power analysis, ensuring a statistical power level of 80%. This increases the statistical reliability of the study despite the small sample size. The small sample size does not compromise the accuracy and precision of the genetic analyses, as a strong molecular method was employed. Furthermore, statistical analyses were conducted appropriately to obtain reliable and valid results. Endometrial tissue samples were collected between the 19th and 21st days of the menstrual cycle. This period is known to be critical for endometrial receptivity. Although individual cyclical variations were not considered in this study, collecting samples during this time window allows for the most consistent assessment of changes in gene expression. The study focused on sampling during a specific window of the menstrual cycle, which represents the period most critical for endometrial receptivity. This limitation could be addressed in future studies by including a broader time window. While the cross-sectional design of the study limits the investigation of changes over time and causal relationships, it does provide valuable insights into the effects of endometrial polyps and myoma uteri on gene expression at a single time point. The study offers an opportunity to examine not only the expression of specific genes but also the molecular impacts of these diseases. Even with a single time point, the study provides precise genetic data analysis, making it a strong contribution. Longitudinal studies can address this limitation, but the current study offers valuable information on how gene expression changes at a specific point in time. The control group consisted of healthy individuals without a diagnosis of endometrial polyps or myoma uteri, allowing for comparisons with the disease groups. Additionally, all participants provided informed consent prior to participation, which strengthens the ethical integrity of the study. The inclusion of healthy individuals in the control group ensures that the study's results can be accurately and reliably compared. Moreover, the proper identification of the patient group and the use of appropriate diagnostic methods allowed for comparability and reliable results with the control group. The study focused on changes in gene expression and examined the molecular effects of diseases such as endometrial polyps and myoma uteri. The lack of consideration of other clinical and lifestyle factors is a limitation that can be addressed in future studies. However, the primary aim of this study was to identify genetic effects. The genetic analyses focused on in this study provide a strong approach to understanding the molecular impacts of endometrial polyp and myoma uteri pathologies, independent of other clinical and lifestyle factors. Future research could include lifestyle and clinical factors, but this study clearly demonstrates the genetic impacts of these diseases.

In conclusion, this study identified a statistically significant increase in the expression of **endometrial PROKR1** in patients diagnosed with endometrial polyps and myoma uteri. Additionally, a significant decrease in the gene expression levels of **PROK1**, **PROKR2**, and **HOXA10** was observed in the endometrial tissue of patients with myoma uteri. These findings align with existing research suggesting a correlation between polymorphisms in the **PROK1** and **PROKR1** genes and recurrent miscarriage. Furthermore, impaired expression levels of **HOXA10** and prokineticins have been associated with various infertility-related conditions, including unexplained infertility, PCOS, endometriosis, and recurrent pregnancy loss. Although the roles of the **HOXA10** gene and prokineticin gene family in infertility are well-recognized, the precise mechanisms by which these genes influence fertility remain poorly understood. Further research is needed to elucidate their roles in normal and pathological implantation processes. Expanding knowledge in this area could offer valuable insights into how conditions like myoma uteri and endometrial polyps impact fertility, potentially leading to novel diagnostic and therapeutic strategies. These findings suggest promising directions for the development of targeted treatments aimed at improving implantation success rates in both natural and assisted reproductive scenarios. In the future, addressing gene expression dysregulation through advanced molecular therapies, such as **gene editing and gene therapy**, may provide more precise and effective treatment options compared to current empirical methods. Such approaches could significantly enhance pregnancy outcomes by mitigating implantation failure, which remains a major cause of infertility, particularly in cases associated with endometrial abnormalities. This study underscores the importance of integrating molecular and clinical perspectives to improve fertility outcomes and provides a foundation for future research in understanding and treating implantation-related infertility.

This study provides important clinical implications for infertility treatments in women diagnosed with endometrial polyps (EP) and myoma uteri (MU). Changes in gene expression could help us better understand the relationship between these conditions and infertility, allowing for the customization of treatment strategies. Given that EP and MU are associated with genetic and molecular factors, genetic-based interventions may play a crucial role in treating these conditions. Specifically, modulation of changes in genes like HOXA10 and the prokineticin gene family could enhance treatment methods. Gene therapy could potentially improve endometrial receptivity and assist with therapeutic interventions. Additionally, genetic testing and molecular profiling could enable personalized treatment approaches. The use of genetic biomarkers to monitor treatment progress could also have a significant impact on assisted reproductive technologies, such as IVF. Ultimately, these findings may contribute to the development of more targeted and effective treatment strategies for women with endometrial polyps and myoma uteri, improving infertility treatments.

## Data Availability

The authors grant permission for the use of data and materials.
